# The antidepressant effect and safety of non-intranasal esketamine: A systematic review

**DOI:** 10.1177/02698811221084055

**Published:** 2022-05-12

**Authors:** Sanne Y Smith-Apeldoorn, Maurice Vischjager, Jolien KE Veraart, Jeanine Kamphuis, Marije aan het Rot, Robert A Schoevers

**Affiliations:** 1 Department of Psychiatry, University Medical Center Groningen, University of Groningen, Groningen, The Netherlands; 2 Department of Mood Disorders, PsyQ Haaglanden, Parnassia Psychiatric Institute, The Hague, The Netherlands; 3 Department of Psychology, University of Groningen, Groningen, The Netherlands

**Keywords:** Esketamine, depression, systematic review

## Abstract

**Background:**

The introduction of esketamine into the field of psychiatry comes on the heels of excitement from studies on racemic ketamine. While the intranasal route has been the most studied to date, other modes of administration of esketamine may also be of interest in the management of depression.

**Aims:**

To systematically review the literature on non-intranasal esketamine for depression in terms of its antidepressant effect and safety.

**Methods:**

We searched PubMed, Embase, the Cochrane Library, and Google Scholar from inception up to February 2021. Search terms included a combination of Medical Subject Headings and text words indicative of esketamine and depression. We selected both controlled and uncontrolled studies examining non-intranasal esketamine for the treatment of depression.

**Results:**

We identified four randomized controlled trials (RCTs) on intravenous esketamine and 15 open-label studies on intravenous (*n* = 80), subcutaneous (*n* = 73), and oral (*n* = 5) esketamine. We found intravenous, subcutaneous, and possibly oral administration of esketamine to be effective in reducing depressive symptoms in most patients with major depressive disorder, bipolar depression, and (severe) treatment-resistant depression. Clinical response to repeated administration of esketamine persisted over the course of treatment. Esketamine was well tolerated by most patients, but open-label data indicate marked psychotomimetic symptoms in exceptional cases. The overall quality of the controlled studies was considered high, the overall quality of the uncontrolled studies low to moderate.

**Conclusions:**

Intravenous, subcutaneous, and possibly oral esketamine may offer an effective and safe addition to the depression treatment armamentarium. However, as most included studies lacked a control group and had small sample sizes, the quality of our results is limited. Different types and formulations of ketamine remain to be compared directly.

## Introduction

In the past two decades, a string of clinical trials and case series has produced relatively consistent evidence of ketamine’s rapid and robust antidepressant effect (Kryst et al., 2020; Marcantoni et al., 2020), offering new hope to patients and their treatment providers. In most studies conducted to date, ketamine has been administered as a racemic mixture comprised of its R-(−)enantiomer (arketamine) and S-(+)enantiomer (esketamine). Both arketamine and esketamine modulate glutamate transmission by acting as N-methyl-D-aspartic acid (NMDA) receptor antagonists. However, the NMDA receptor binding affinity of esketamine is three to four times higher than that of arketamine (Kohrs and Durieux, 1998). As ketamine’s antidepressant properties are mostly believed to stem from ketamine’s impact on glutamate neurotransmission (Abdallah et al., 2018), esketamine should theoretically yield the better therapeutic effect.

In recent years, a growing number of studies have investigated the antidepressant effect of esketamine. The intranasal (IN) route has been the most studied mode of administration of this enantiomer. Studies have shown IN esketamine to be an effective treatment strategy for patients with major depressive disorder (MDD) who are treatment-resistant or acutely suicidal. Recent meta-analyses covering more than 700 patients show that IN esketamine is associated with significantly higher response and remission rates starting at 2 h, peaking at 24 h, and at least lasting for 28 days, with risk ratios of response and remission at day 28 of 1.36–1.38 and 1.38–1.42, respectively (Papakostas et al., 2020; Zheng et al., 2020). In addition, maintenance treatment with IN esketamine may be associated with stable efficacy in relapse prevention (Daly et al., 2018, 2019; Wajs et al., 2020). Based on these results, in 2019, the United States Food and Drug Administration and the European Medicines Agency approved an esketamine nasal spray for adults with treatment-resistant depression (TRD). However, the efficacy of the nasal spray has also been questioned, (Horowitz and Moncrieff, 2020; Schatzberg, 2019; Turner, 2019) and IN esketamine may have adverse effects that are specific to its mode of administration (e.g. taste disturbance, postnasal drip, and stuffy nose; Wajs et al., 2020). In addition, the current price of the IN spray might limit its widespread use among patients with TRD (Ross and Soeteman, 2020). Formulations of esketamine other than IN could, therefore, also be of interest in the management of depression. Non-intranasal esketamine has been studied as intravenous (IV), oral, and subcutaneous (SC) formulations, but results of these studies have not yet been synthesized as the results of IN esketamine have been. The aim of this systematic review was to provide an overview of studies on the antidepressant effect and safety of non-intranasal esketamine in the treatment of depression.

## Method

This review was conducted and reported according to the Preferred Reporting Items for Systematic Meta-Analyses (PRISMA) guidelines (Moher et al., 2009). Its methods were preregistered (PROSPERO, CRD42020209666).

### Search strategy and selection criteria

We searched PubMed, Embase, the Cochrane Library, and Google Scholar from inception to 9 February 2021. Search terms included a combination of Medical Subject Headings and text words indicative of (1) esketamine and (2) depression. The full search strings are given in Supplementary Table 1. No restrictions were set when searching the databases. Database search and eligibility assessment were performed independently in a standardized manner by two reviewers (SYS-A and MV**)**. Disagreements were generally resolved through consensus; persistent disagreement regarding the eligibility of patient populations was resolved by an arbitrator (JKEV) twice. A log was kept with excluded articles and reasons for exclusion. Reference lists of included articles were hand-searched to identify additional relevant publications.

Following the participants, intervention, comparison, outcomes, and study design (PICOS) strategy, we included studies for which the following criteria were met: (1) Participants: men and women of any age with any type of depression, including bipolar depression; (2) Intervention: treatment with non-intranasal esketamine, regardless of dose, duration, and frequency; (3) Comparison: any control intervention or no control intervention; (4) Outcomes: (a) Antidepressant effect, as defined by depressive symptom reduction measured by validated questionnaires, clinician-observed or patient-reported reduction in depressive symptoms, response rates, or remission rates; (b) Safety, as defined by adverse events, serious adverse events, or discontinuation due to adverse events; (5) Study design: controlled and uncontrolled studies, including randomized controlled trials (RCTs), pre-post studies, cohort studies, case series, and case reports. While RCTs are thought to provide the highest level of evidence, other study designs may also provide important information, particularly in the emerging field of esketamine for depression. We, therefore, included both controlled and uncontrolled studies. Letters or comments to editors were included if they reported on original data (e.g. case series). Only articles in English, Dutch, or German were included.

### Data collection and analysis

We extracted data on study design and setting, source of funding, participant inclusion and exclusion criteria, sample characteristics (demographics and clinical data), esketamine intervention details (route, dose, and number of doses), comparison intervention details (type, route, dose, and number of doses), and outcome (instruments, timing, and results). We also inventoried authors’ conclusions. Data were extracted by one author (SYS-A) and checked by a second author (MV), using a pilot-tested data extraction form. Disagreements were resolved through consensus. To confirm unclear data, corresponding authors were contacted.

Two independent reviewers (SYS-A and MV) assessed bias of the included studies by the use of the Cochrane risk-of-bias tool for randomized trials, the Newcastle-Ottawa Scale for case–control studies, and the Quality Appraisal of Case Series Studies Checklist for case reports and case series. Disagreements were resolved through consensus.

A meta-analysis of RCT data was initially planned but deemed inappropriate after data extraction. The search revealed only four RCTs and these were too diverse to pool in terms of study group heterogeneity (see Table 1). Therefore, a qualitative systematic review of both the open-label study data and RCT data was undertaken.

**Table 1.** Characteristics of included randomized controlled trials.

Author Year Country	Main selection criteria	Intervention details	Study groups	Antidepressant effects	Safety
Correia-Melo et al. (2020) Vieira et al. (2021) Brazil	Inclusion: MDD TRD (⩾ 1 AD) Exclusion: Recent ECT Psychotic disorder	Esketamine: Route: IV (40 min) Dose: 0.25 mg/kg No: single Racemic ketamine: Route: IV (40 min) Dose: 0.5 mg/kg No: single Co-intervention: Ongoing AD was maintained	Esketamine: *N*: 34 Female: 56% Age: 45.5 (±14.5) Episodes: 8.0 (±6.5) Duration CE (mos): 32.9 (±65.7) Therapeutic failures ⩾ 3: 56% Baseline MADRS: 33.1 (±9.3) Racemic ketamine: *N*: 29 Female: 70% Age: 48.7 (±15.1) Episodes: 5.9 (±5.7) Duration CE (mos): 24.9 (±43.5) Therapeutic failures ⩾ 3: 65% Baseline MADRS: 32.9 (±5.3)	MADRS scores esketamine vs ketamine: 24 h: 17.5 vs 16.2 (*p* = 0.67) 72 h: 17.4 vs 14.9 (*p* = 0.44) 7 days: 20.6 vs 14.3 (*p* = 0.08) Response esketamine vs ketamine: 24 h: 50% vs 52% (95% CILB −22.5) 72 h: 48% vs 57% (95% CILB −30.1) 7 days: 44% vs 62% (95% CILB −39.0) Remission esketamine vs ketamine: 24 h: 29% vs 24% (95% CILB −13.6) 72 h: 36% vs 39% (95% CILB −24.6) 7 days: 28% vs 41% (95% CILB −33.2) Suicidality esketamine vs ketamine: Baseline: 2.0 vs 2.0 (*p* = 0.27) 24 h: 0.0 vs 0.0 (*p* = 0.89) 7 days: 0.0 vs 0.0 (*p* = 0.14)	CADSS score esketamine vs ketamine: During infusion: 14.9 vs 18.2 (*p* = 0.45) Most common TEAE: ↑ BP ↑HR Nausea Dissociation SAE: none Drop-out: none
Liu et al. (2020) China	Inclusion: Breast cancer Mastectomy HDRS_17_ 8–24 Exclusion: Psychiatric comorbidity Psychiatric history	Esketamine: Route: IV^a^ Dose: 0.125 mg/kg No: single Racemic ketamine: Route: IV^a^ Dose: 0.125 mg/kg No: single Placebo (saline): Route: IV^a^ No: single	Esketamine: *N*: 101 Age: 46.6 (±8.2) Baseline HDRS_17_: 16.8 (±2.3) Racemic ketamine: *N*: 102 Age: 47.7 (±9.7) Baseline HDRS_17_: 17.0 (±2.2) Placebo: *N*: 100 Age: 48.0 (±10.2) Baseline HDRS_17_: 17.0 (±2.2)	HDRS_17_ scores esketamine vs ketamine: 3 days: 11.4 vs 13.2 (*p* < 0.05) 1 week: 9.4 vs 10.5 (*p* < 0.05) 1 month: 6.9 vs 9.5 (*p* < 0.05) 3 months: 6.5 vs 7.5 (NS) HDRS_17_ scores esketamine vs placebo: 3 days: 11.4 vs 16.4 (*p* < 0.05) 1 week: 9.4 vs 11.2 (*p* < 0.05) 1 month: 6.9 vs 11.0 (*p* < 0.05) 3 months: 6.5 vs 7.5 (NS)	AE esketamine vs ketamine vs placebo: Nausea: 16% vs 18% vs 20% (NS) Dizziness: 12% vs 11% vs 13% (NS) Vomiting: 7% vs 8% vs 7% (NS)
Singh et al. (2016) Lewis et al. (2019) Belgium Poland Germany	Inclusion: Recurrent MDD TRD (⩾ 2 AD) IDS-C ⩾ 34 Exclusion: Psychotic features Recent suicidality requiring hospitalization Previous ketamine nonresponse	Phase 1 (DB): Esketamine: Route: IV (40 min) Dose: 0.2/0.4 mg/kg No: 1–2 in 4 days Placebo (saline): Route: IV (40 min) No: 1–2 in 4 days Phase 2 (open-label):	Esketamine (0.20 mg/kg): *N*: 9 Female: 56% Age: 44.7 (±13.4) Therapeutic failures ⩾ 4: 33% Baseline MADRS: 33.1 (±3.6) Esketamine (0.40 mg/kg):	∆ MADRS day 2: Placebo vs esketamine 0.20 mg/kg: −3.8 vs −16.8 (*p* = 0.001, Cohen’s *d* = −1.54) Placebo vs esketamine 0.40 mg/kg: −3.8 vs −16.9 (p = 0.001, Cohen’s *d* = −1.70) Response day 2:	AE ⩾ 1 and TEAE: Placebo: 50% and 30% Esketamine 0.20 mg/kg: 50% and 25% Esketamine 0.40 mg/kg^2^: 70% and 67% Most common AE placebo vs esketamine 0.20 mg/kg vs esketamine 0.40 mg/kg^b^: Dissociation: 0% vs 8% vs 17%
		Esketamine: Route: IV (40 min) Dose: max 0.4 mg/kg No: max 4 in 11 days	*N*: 11 Female: 64% Age: 41.8 (±11.6) Therapeutic failures ⩾ 4: 64% Baseline MADRS: 33.7 (±5.8) Placebo: *N*: 10 Female: 60% Age: 42.7 (±10.9) Therapeutic failures ⩾ 4: 70% Baseline MADRS: 33.9 (±4.2)	Placebo vs esketamine 0.20 mg/kg: 0% vs 67% (OR 40.2, *p* = 0.013) Placebo vs esketamine 0.40 mg/kg: 0% vs 64% (OR 34.5, *p* = 0.014) ∆ MADRS at follow-up: Day 17 (end of open-label treatment): − 17.0 (±12.77) to −26.0 (±9.59) Day 35 (end of follow-up): −15.7 (±8.51) to −25.0 (±14.54) Exit interview blinded analyses: Improved mood: *n* = 13 ↑ activities: *n* = 7 Improved cognition: *n* = 6 ↑ energy: *n* = 5	Dizziness: 0% vs 8% vs 3% Dry mouth: 0% vs 8% vs 7% Headache: 20% vs 17% vs 23% Nasopharyngitis: 0% vs 0% vs 7% Nausea: 20% vs 25% vs 10% Oropharyngeal pain: 0% vs 8% vs 3% Paresthesia: 0% vs 0% vs 7% Rash: 0% vs 8% vs 0% Thrombophlebitis: 0% vs 8% vs 0% Tooth infection: 10% vs 0% vs 0% Vertigo: 0% vs 0% vs 7% Vomiting: 0% vs 8% vs 3% Mean BPRS and CADSS total score: Max: 30–40 min after start infusion Dose-related Return to baseline level: ⩽ 2 h and ⩽ 4 h Vital sign abnormalities esketamine groups^b^: Irregular breathing: *n* = 1 Transient high BP: *n* = 1 SAE^b^: *n* = 1 Drop-out D/T AE^b^: *n* = 1
Wang et al. (2020) China	Inclusion: Cervical carcinoma Hysterectomy HDRS_17_ 8–24 Exclusion: Psychiatric comorbidity	Esketamine: Route: IV^c^ Dose: 0.25/0.5 mg/kg No: single Racemic ketamine: Route: IV^c^ Dose: 0.5 mg/kg No: single Placebo (saline): Route: IV^c^ No: single	Esketamine (0.25 mg/kg): *N*: 104 Age: 48.1 (±10.4) Baseline HDRS_17_: 16.7 (±5.0) Esketamine (0.5 mg/kg): *N*: 104 Age: 48.5 (±10.0) Baseline HDRS_17_:15.8 (±4.6) Racemic ketamine: *N*: 104 Age: 47.1 (±10.1) Baseline HDRS_17_: 16.2 (±4.9) Placebo: *N*: 105 Age: 46.3 (±10.8) Baseline HDRS_17_: 15.8 (±4.8)	HDRS_17_ scores esketamine 0.5 mg/kg vs esketamine 0.25 mg/kg, ketamine and placebo: 1 day: *p* < 0.05, *p* < 0.05, *p* < 0.05 2 days: *p* < 0.05, *p* < 0.05, *p* < 0.05 3 days: *p* < 0.05, *p* < 0.05, *p* < 0.05 5 days: NS, NS, NS 7 days: NS, NS, NS HDRS_17_ scores esketamine 0.25 mg/kg vs ketamine and placebo: 1 day: NS, *p* < 0.05 2 days: NS, *p* < 0.05 3 days: NS, *p* < 0.05 5 days: NS, NS 7 days: NS, NS	AE esketamine 0.25 mg/kg vs esketamine 0.50 mg/kg vs ketamine vs placebo: Nausea: 16% vs 18% vs 19% vs 17% (NS) Dizziness: 12% vs 13% vs 13% vs 11% (NS) Vomiting: 8% vs 9% vs 10% vs 8% (NS)

MDD: major depressive disorder; TRD: treatment-resistant depression; ECT: electroconvulsive therapy; IV: intravenous; AD, antidepressant; CE: current episode; MADRS: Montgomery–Åsberg Depression Rating Scale; CILB: confidence interval lower bound; CADSS: Clinician Administered Dissociative States Scale; TEAE: treatment-emergent adverse event; BP: blood pressure; HR: heart rate; SAE: serious adverse event; HDRS: Hamilton Depression Rating Scale; NS: not significant; AE: adverse event; IDS: Inventory of Depressive Symptomatology; DB: double blind; BPRS: Brief Psychiatric Rating Scale.

a After analgesia induction.

b Combined DB and open-label phases.

c 1 h after analgesia.

## Results

### Study selection

**Figure 1.**
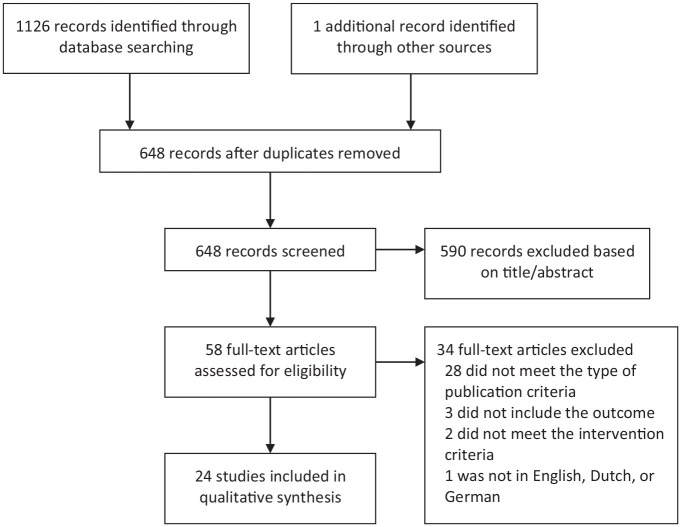
PRISMA flowchart for study selection.

Overall, 1126 records were identified through database search. One additional record was identified by the hand search of reference lists. After adjusting for duplicates, 648 records remained. Of these, 590 were discarded after reviewing titles and abstracts. Of the remaining 58 full-text articles, 24 met the inclusion criteria (Ajub and Lacerda, 2018; Barbosa et al., 2020; Bartova et al., 2018, 2015; Correia-Melo et al., 2017a, 2017b, 2020; Del Sant et al., 2020; Delfino et al., 2021; Falk et al., 2020; Findeis et al., 2020; Kallmünzer et al., 2016; Kavakbasi et al., 2021; Lewis et al., 2019; Liu et al., 2020; Lucchese et al., 2021; Paslakis et al., 2010; Paul et al., 2009; Ritter et al., 2020; Segmiller et al., 2013; Singh et al., 2016; Veraart et al., 2021a; Vieira et al., 2021; Wang et al., 2020). Nine articles included the same four cohorts of patients. Furthermore, two reports appear to have included two overlapping patients. Nonetheless, since all 11 articles provided complementary results, all were included in the systematic review. Only original and novel results were presented. Patients were counted once, except for the two patients of whom overlap was not confirmed. The steps involved in the selection of studies are illustrated in a flowchart given in Figure 1.

### Study characteristics

We included four RCTs (Table 1), 14 case series and reports (Table 2), and one retrospective case–control study (Table 2), with a total of 981 patients. Most studies included patients with a depressive episode in the course of MDD or bipolar disorder (BD) and some degree of treatment resistance. In four studies, besides meeting criteria for depression, patients underwent palliative care or cancer treatment (Barbosa et al., 2020; Falk et al., 2020; Liu et al., 2020; Wang et al., 2020). The severity of depressive symptoms was mostly measured by the Montgomery–Åsberg Depression Rating Scale (MADRS) or Hamilton Depression Rating Scale (HDRS). Adverse events were mostly measured by a questionnaire on dissociative symptoms (that is, the Clinician Administered Dissociative States Scale—CADSS), vital signs, clinician observations, or subject reports. The timing of the outcome measurements was variable (i.e. repeated evaluations until end-of-treatment, follow-up evaluations up to 3 months, or a single end-of-treatment evaluation).

**Table 2.** Characteristics of included open-label trials.

Author Year Country Design	Intervention details	Sample	Antidepressant effects	Safety
Ajub and Lacerda (2018) Brazil Case series	Esketamine: Route: IV (40 min) or SC Dose: 0.5 mg/kg No: single Co-intervention: Ongoing AD was maintained	Esketamine: *N*: 3^a^ Female: 3 Age: 41, 44, 45 Diagnosis: MDD (*n* = 2) or BD (*n* = 1) with psychotic features Comorbidity: alcohol dependence (*n* = 2), social anxiety disorder (*n* = 1) Baseline MADRS: 36, 42, 55	∆MADRS at 24 h: Subject 1: –39 (55 to 16) Subject 2: –34 (36 to 2) Subject 3: –35 (42 to 7) Subject report at follow-up: Subject 1: mild depressive symptoms Subject 2: remission Subject 3: remission	AE: Dissociative symptoms: *n* = 2 Nausea: *n* = 1 Light-headedness: *n* = 1
Barbosa et al. (2020) Brazil Case report	Esketamine: Route: SC Dose: 0.5–0.75 mg/kg No: 4 in 9 days	Esketamine: *N*: 1 Female: 0 Age: 65 Diagnosis: MDD Comorbidity: abdominal tumor Episodes: 1 Therapeutic failures: none Baseline MADRS: 30	MADRS scores: Day 2 (24 h post first injection): 20 Day 3 (pre-second injection): 18 Day 4 (24 h post second injection): 17 Day 6 (pre third injection): 21 Day 7 (24 h post third injection): 9 Day 9 (pre fourth injection): 10 Day 10 (24 post fourth injection): missing D/T somnolence and respiratory distress Subject report at day 4: Felt well, cheerful, and had “strength to continue”	CADSS score at 30, 60, 90 min post injection: Day 1: 0, 0, 0 Day 3: 37, 3, 0 Day 6: 9, 3, 0 Day 9: missing D/T somnolence, 20, 0 Vital parameters (max variations): BP: 11 mmHg systolic, 19 mmHg diastolic HR: 10 BPM Oximetry: 3% AE: ↑ abdominal pain (day 6) Respiratory distress (day 6–9) Somnolence (day 9)
Bartova et al. (2015) Austria Case series	Esketamine: Route: IV Dose: 50 mg (0.85 mg/kg) or 75 mg (0.63 mg/kg) No: “repeated” Co-intervention: Tranylcypromine	Esketamine: *N*: 2 Female: 2 Age: 43, 74 Diagnosis: TRD with suicidal crisis Therapeutic failures: “multiple”	Clinician observed: Subject 1: good anti-suicidal effects Subject 2: good anti-suicidal effects	Vital parameters: Subject 1: no relevant changes according to authors Subject 2: stable according to authors Drop-out: none
Bartova et al. (2018) Austria Case report	Esketamine: Route: IV (30 min) Dose: 37.5 mg (0.33 mg/kg) Frequency: thrice weekly Duration: 3 weeks Co-intervention: Ongoing AD was maintained	Esketamine: *N*: 1 Female: 1 Age: 30 Diagnosis: Post-psychotic depression Baseline MADRS: 48	MADRS scores: After first treatment: 6 End of treatment: 4	PANSS-P score: After first treatment: 8 End of treatment: 7 CADSS score: During first treatment: 16 Return to baseline level: ⩽ minutes Vital parameters: No relevant changes according to authors
Correia-Melo et al. (2017a) Brazil Case series	Esketamine: Route: IV (10 min) Dose: 0.25 mg/kg No: single Co-intervention: Ongoing AD was maintained	Esketamine: *N*: 27 Female: 39% Age: 51 (42–64) Diagnosis: MDD (85%) or BD (15%) Episodes: 4.0 (2.8–6.0) Duration CE: “chronic in majority” Baseline MADRS: 36.3 (±7.6)	MADRS scores and change: 24 h: 17.4 (±14.7), ∆: −18.7 (±2.3) (*p* < 0.001) 72 h: 18.7 (±15.5), ∆: −17.5 (±2.3) (*p* < 0.001) 7 days: 19.0 (±14.3), ∆: −17.2 (±2.3) (*p* < 0.001) Response and remission: 24 h: 59% and 41% 72 h: 52% and 37% 7 days: 48% and 37%	Vital signs, ECG, clinical laboratory assessments: Within normal ranges according to authors Mild to severe dissociative symptoms: 11% Drop-out/lost to follow-up: *n* = 4
Correia-Melo et al. (2017b) Brazil Case series	Esketamine: Route: IV (10 min) Dose: 0.25 mg/kg No: single Co-intervention: Ongoing AD was maintained	Esketamine: *N*: 2 Female: 2 Age: 43, 66 Diagnosis: TRD Therapeutic failures: 3 AD + ⩾ 2 augmentation trials Baseline MADRS: 40, 48	∆ MADRS at 24 h: Subject 1: −12 (40 to 28) Subject 2: −17 (48 to 31) Subject report at 3 weeks follow-up: Subject 1: Remission	Subject report: Subject 1: marked dissociative symptoms—terrible experience Subject 2: traumatic dissociative symptoms Subject report at follow-up: Subject 1: re-experiences of dissociative thoughts and nightmares. Remission at 3 weeks. Subject 2: persistent dissociative and psychotic behavior. Remission at 4 weeks.
Del Sant et al. (2020) Delfino et al. (2021) Lucchese et al. (2021) Brazil Case series	Esketamine: Route: SC Dose: 0.5–1.0 mg/kg Frequency: weekly Duration: 6 weeks Co-intervention: Ongoing AD was maintained	Esketamine: *N*: 70 Female: 64% Age: 40.3 (±12.7) Diagnosis: MDD (56%) or BD (44%) Comorbidity (anxiety): 44% Duration CE chronic: 70% Therapeutic failures ⩾ 5: 80% Augmentation failures: 90% Baseline MADRS: 33.6 (±6.3)	Response and remission: Day 42: 50% and 26% ∆ Anhedonia (MADRS item 8): 24 h: *t* = 4.007 (*p* < 0.001) Day 42: *F* = 5.827 (*p* < 0.0001) Time×diagnosis interaction: *F* = 1.099 (*p* = 0.379)	Vital functions: ↑ SBP > 30 mmHg and ↑ DBP > 15 mmHg: 30% SBP ⩾ 180 mmHg and/or DBP ⩾ 110 mmHg: 20% Return to pretreatment levels: ⩽ 120 min post dose Drop-out D/T cardiovascular side effects: none Deaths: none Drop-out: Del Sant et al.: 10% Delfino et al.: 16% Lucchese et al.: 9%
Falk et al. (2020) Germany Case–control, retrospective	Esketamine: Route: IV (45 min) Dose: 0.25 mg/kg No: unknown	Esketamine: *N*: 8 Female: 4 Age: 52.1 (± 13.3) Baseline AD: 75% Baseline STADI anxiety: 68.9 (±11.0) Baseline STADI depress: 66.4 (±10.9) Control: *N*: 8 Female: 3 Age: 54.6 (±13.2) Baseline AD: 38% Baseline STADI anxiety: 57.4 (±13.4) - Baseline STADI depress: 59.3 (±12.5)	STADI depression scores control vs esketamine: Day 1 – 5: 59.0 (±13.4) vs 57.8 (±12.8) Test statistics group: 0.31 (*p* = 0.59) Test statistics time: 1.80 (*p* = 0.20) Test statistics group×time: 1.60 (*p* = 0.23)	Restlessness and anxiety (PSBS) T = 0, z = −1.00 (*p* = 0.32)
Findeis et al. (2020) Ritter et al. (2020) Germany Case series	Esketamine Route: IV (60 min) + subsequent SC Dose: 0.25–0.5 mg/kg Frequency: 2–3 weekly Duration: unknown No of administrations: Findeis et al.: 1–34 Ritter et al.: 1–8 Co-intervention: Ongoing AD was maintained	Findeis et al. (2020): *N*: 25 Female: 60% Age: 49 (±15) Diagnosis: MDD (64%), BD (28%) or SD (8%) Comorbidity: “Emotionally instable personality disorder” (16%), alcohol misuse (16%), PTSD (8%), somatoform disorder (4%) Baseline BDI: 30.9 (±13.3)	BDI score post treatment: 20.9 (± 13.8) (*p* < 0.001) Response post treatment: Intention to treat: 31% Per protocol: 38% Remission post treatment: Intention to treat: 45% Per protocol: 54%	AE: Transient BPS > 200: *n* = 1 Intrusion like negative memories: *n* = 4 Increased anxiety: *n* = 2 Transient confusional state: *n* = 1 Drop-out D/T AE: Findeis et al.: 8% Ritter et al.: 17% Urothelial toxicity: Leukocyte concentration: *F* = 3.1 (*p* = 0.2)
		Ritter et al. (2020): *N*: 29 Diagnosis: MDD (66%) or BD (34%)		Erythrocyte concentration: *F* = 4.1 (*p* = 0.2) Protein: no ↑ in detectable levels Free hemoglobin: no ↑ in detectable levels
Kallmünzer et al. (2016) Germany Case series	Esketamine Route: IV (45 min) Dose: 0.3 mg/kg No: 7 in 10 weeks Co-intervention: 12 weekly ECT sessions in alternating sequences Ongoing AD was maintained	Esketamine: *N*: 3 Female: 1 Age: 63, 65, 73 Diagnosis: MDD (*n* = 2) or BD (*n* = 1) Comorbidity: PTSD (*n* = 1), CPD (*n* = 1) Duration CE (weeks): 7, 9, 16 Therapeutic failures: ⩾ 4 AD and ECT Baseline HADS: 12, 37, 50 Baseline BDI: 16, 38, 50	Response: Week 3: *n* = 1 Week 9: *n* = 3 Remission: Week 5: *n* = 1 Week 10: *n* = 2 4-week follow-up: *n* = 3	∆ MMST at discharge: Subject 1: 0 (29 29) Subject 2: –1 (27 26) Subject 3: 2 (26 28) AE (clinician observed/subject report): Upper respiratory infection Transient worsening lower back pain Carious tooth burst while receiving ECT anesthesia Recurrent headaches
Kavakbasi et al. (2021) Germany Case report	Esketamine Route: IV Dose: 1.0 mg/kg No: 4 in 18 days Co-intervention: 6 thrice weekly ECT sessions in alternating sequences Ongoing AD was maintained	Esketamine: *N*: 1 Female: 1 Age: 56 Diagnosis: TRD Duration CE (months): 6 Therapeutic failures CE: AD, ECT, IV esketamine 0.75 mg/kg (9 infusions) Baseline MADRS: 36	MADRS score: End of treatment: 9	AE (clinician observed/subject report): Mild disorientation, which subsided after discontinuation of lithium Well-tolerated without any relevant complications
Paslakis et al. (2010) Germany Case series	Esketamine: Route: oral Dose: 1.25 mg/kg Frequency: daily Duration: 12–14 days Co-intervention: Venlafaxine Duloxetine	Esketamine: *N*: 4 Age: 36, 42, 51, 57 Diagnosis: MDD Comorbidity: alcohol abuse (*n* = 1) Duration CE (months): 2–60 Therapeutic failures: none—“several” Baseline HDRS: 19, 21, 24, 24	∆ HDRS at 7 days and 14 days: Subject 1: −12 and −16 (24 to 12 to 8) Subject 2: −1 and −5 (24 to 23 to 19) Subject 3: 1 and −4 (19 to 20 to 15) Subject 4: −13 and −13 (21 to 8 to 8) BDI scores: Scores corresponded well to the HDRS scores according to authors	AE (clinician observed/subject report): Well-tolerated Essentially no side effects No psychomimetic effects
Paul et al. (2009) Germany Case series, cross-over	Esketamine: Route: IV (50 min) Dose: 0.25 mg/kg No: single Racemic ketamine: Route: IV (50 min) Dose: 0.5 mg/kg No: single Co-intervention: Ongoing AD was maintained	Esketamine: *N*: 2 Female: 1 Age: 51, 58 Diagnosis: MDD Comorbidity: none Episodes: 3, 6 Therapeutic failures: 8, 11 Baseline HDRS_21_: 24, 26	HDRS_21_ scores subject 1 esketamine vs ketamine: Baseline: 24 vs 25 1 h: 24 vs 25 1 day: 25 vs 25 3 days: 24 vs 25 6 days: 25 vs 25 HDRS_21_ scores subject 2 esketamine vs ketamine: Baseline: 25 vs 26 1 h: 25 vs 26 1 day: 14 vs 11 3 days: 15 vs 11 6 days: 24 vs 25	Subject report subject 1: Esketamine: fatigue, “muzzy” Racemic ketamine: sensation that walls were moving, unintentionally crying Subject report subject 2: Esketamine: tiredness Racemic ketamine: dizziness, “embedded”, colors with “whiff of pink” Cardiovascular complications: None according to authors
Segmiller et al. (2013) Germany Case series	Esketamine Route: IV (40 min) Dose: 0.25 mg/kg No: 6 in 4 weeks Co-intervention: Ongoing AD was maintained	Esketamine: N: 6 Female: 3 Age 58.8 (±19) Diagnosis: MDD Duration CE (weeks): 22.7 (16–36) Therapeutic failures: ⩾ 2 AD Baseline HDRS_21_: 24.8 (19–35)	∆ HDRS_21_ pre- and post-final infusion: Subject 1: –5 (19 to 14) and –8 (19 to 11) Subject 2: –20 (22 to 2) and –20 (22 to 2) Subject 3: –7 (19 to 12) and –9 (19 to 10) Subject 4: –9 (35 to 26) and –10 (35 to 25) Subject 5: –17 (21 to 4) and –19 (21 to 2) Remission: Post treatment: 33%	Pronounced to severe dissociative symptoms: *n* = 2 Drop out D/T dissociative symptoms: *n* = 1
Veraart et al. (2021a) Netherlands Case report	Esketamine: Route: oral Dose: 2.0 mg/kg Frequency: twice weekly Duration: 18 months Co-intervention: Ongoing AD was maintained DBS settings were kept stable	Esketamine: *N*: 1 Female: 1 Age: 55 Diagnosis: TRD with psychotic features Comorbidity: OCD Therapeutic failures: ⩾ 4 AD, augmentation, psychotherapy, ECT, DBS Baseline HDRS_17_: 24	∆ HDRS_17:_ 6 weeks: −18 (24 to 6) ∆ IDS-SR:_:_ 6 weeks: −24 (54 to 30) Clinician observed/subject report: ↑ functioning in important domains of life ↓ auditory hallucinations Remission: 18 months follow-up: *n* = 1	Vital parameters: Stable according to authors AE: Temporary dizziness

AD: antidepressant; MDD: major depressive disorder; AE: adverse events; MADRS: Montgomery–Åsberg Depression Rating Scale; SC: subcutaneous; CADSS: Clinician Administered Dissociative States Scale; BP: blood pressure; HR: heart rate; TRD: treatment-resistant depression; PANSS-P: Positive and Negative Syndrome Scale—Positive Symptoms Subscale; ECG: electrocardiogram; BD: bipolar depression; CPD: chronic pain disorder; DBP: diastolic blood pressure; SBP: systolic blood pressure; STADI: State Trait Anxiety Depression Inventory; SD: schizoaffective disorder; PSBS: Palliative Symptom Burden Score; PTSD: post-traumatic stress disorder; HDRS: Hamilton Depression Rating Scale; IV: intravenous; BDI: Beck Depression Inventory; ECT: electroconvulsive therapy; HADS: Hospital Anxiety and Depression Scale; MMST: Mini Mental State Examination; CE: current episode; DBS: deep brain stimulation; OCD: obsessive-compulsive disorder; IDS: Inventory of Depressive Symptomatology.

a A fourth patient is excluded from this review as his primary diagnosis was SD.

Treatment regimens varied between: single IV infusion (*n* = 10), up to nine repeated IV infusions (*n* = 6), single SC injection (*n* = 1), up to 34 repeated SC injections (*n* = 3), and up to approximately 150 repeated oral administrations (*n* = 2). Esketamine dosages ranged from single to thrice weekly 0.125 to 1.0 mg/kg IV administration, and from single to thrice weekly 0.25 to 1.0 mg/kg SC administration. Oral esketamine was administered in dosages of 1.25 mg/kg daily or 2.0 mg/kg twice weekly. In most studies, patients continued to take their antidepressant medication. In two studies, esketamine administration was alternated with ECT (Kallmünzer et al., 2016; Kavakbasi et al., 2021).

### Quality assessment

The overall quality of the RCTs and the case–control study was considered high. The overall quality of the case reports and series was considered low to moderate, with most case reports and series having a high risk of bias in several domains. More details are provided in Supplementary Tables 2 to 4.

### Antidepressant effects

#### RCT results

Singh et al. (2016) compared a single 0.20 or 0.40 mg/kg 40-min IV infusion of esketamine to placebo (saline) in 30 patients with recurrent MDD. Mean MADRS decrease from baseline to day 2 was significant for both esketamine groups compared to placebo. Moreover, esketamine participants met responder criteria in 67% and 64%, respectively, while there were no responders among placebo participants. These results indicate substantial efficacy of esketamine in either a lower or higher subanesthetic dose.

A second RCT was performed in 63 patients with MDD. This involved a comparison between a single 40-min IV infusion of esketamine (0.25 mg/kg) and racemic ketamine (0.5 mg/kg). Mean MADRS decrease from baseline to day 2 was comparable between the two groups. Responder and remission rates were 50% and 29% for the esketamine group and 52% and 24% for the ketamine group, respectively, confirming non-inferiority. There was a trend toward a more prolonged antidepressant effect over the 7-day follow-up of racemic ketamine, but the difference was not statistically significant (Correia-Melo et al., 2020; Vieira et al., 2021).

Two RCTs compared a single IV injection of esketamine to both racemic ketamine and placebo (saline) in patients with cancer and mild-to-moderate depressive symptoms (Liu et al., 2020; Wang et al., 2020). In the study by Liu et al. (2020), HDRS_17_ scores were lower after 0.125 mg/kg esketamine compared to 0.125 mg/kg racemic ketamine and placebo at 3 days, 1 week, and 1 month follow-up, but not at 3 months follow-up. Similar results were obtained when comparing 0.5 mg/kg esketamine to 0.5 mg/kg racemic ketamine and placebo. However, when comparing 0.25 mg/kg esketamine to both 0.5 mg/kg racemic ketamine and placebo, HDRS_17_ scores were only lower when compared to placebo (Wang et al., 2020). These results suggest that esketamine improves depressive symptoms at the short term in patients with cancer and mild-to-moderate depressive symptoms, and that the effects are better than with the same dose of racemic ketamine.

Overall, these results indicate a rapid onset of antidepressant effects in depressed patients after a single IV infusion of esketamine. Besides, they suggest that esketamine is at least comparable to racemic ketamine on the short term (i.e. up to 3 days). Differences on the longer term (i.e. 7 days and up) are not conclusive. More details are provided in Table 1.

#### Results of open-label studies

##### Single IV infusion

The antidepressant effect of a single IV infusion of esketamine was the topic of three case series and one retrospective case–control study (Ajub and Lacerda, 2018; Correia-Melo et al., 2017a; Falk et al., 2020; Paul et al., 2009).

Correia-Melo et al. (2017a) described 27 patients with MDD or BD who were treated with a rapid, 10 min infusion of 0.25 mg/kg esketamine. This resulted in response and remission rates of 59% and 41% at 24 h follow-up, 52% and 37% at 72 h follow-up, and 48% and 37% at 7 days follow-up.

Of the three patients with depression with psychotic features described by Ajub and Lacerda (2018), one received 0.5 mg/kg infusion of esketamine over 40 min. This resulted in remission of suicidal ideation and psychotic features at 24 h, which was maintained for up to 2 weeks follow-up.

Paul et al. (2009) reported on two MDD patients consecutively treated with 0.5 mg/kg racemic ketamine and 0.25 mg/kg esketamine administered IV over 50 min. One patient did not respond to either treatment, the other patient responded to both.

In the retrospective case–control study by Falk et al. (2020), data from 16 palliative-care inpatients were analyzed. For analgesic purposes, eight patients had received treatment with 0.25 mg/kg esketamine IV infusion over 45 min. The other eight patients did not need pain control and were therefore not treated with esketamine. Depressive symptom reduction did not differ between the two subgroups.

In summary, these results suggest a rapid onset of antidepressant effects of a single IV infusion of esketamine in patients with MDD or BD, but not in palliative care patients.

##### Repeated IV infusions

Data relating to repeated IV infusions of esketamine were available from six studies published in seven articles (Bartova et al., 2018, 2015; Kallmünzer et al., 2016; Kavakbasi et al., 2021; Lewis et al., 2019; Segmiller et al., 2013; Singh et al., 2016).

First, a case series of six MDD patients demonstrated improvement in three patients and remission in two patients, both at the short term (i.e. hours to days) and over the course of the 4-week 40-min 0.25 mg/kg esketamine treatment (Segmiller et al., 2013).

Bartova et al. (2015, 2018) treated two MDD patients with acute severe suicidality and one patient with post-psychotic depression. They described “good anti-suicidal effects” and “sustained remission of depression and suicidality” during treatment with repeated 30-min IV infusions of up to 0.85 mg/kg esketamine.

The results of a post-RCT open-label treatment with up to four 40-min IV infusions of 0.40 mg/kg esketamine are in line with the results of these case series. Specifically, results showed improvement at the end of treatment and at 35 days follow-up (Lewis et al., 2019; Singh et al., 2016).

Kallmünzer et al. (2016) and Kavakbasi et al. (2021) reported on a novel therapeutic regimen combining repeated esketamine IV infusions and ECT sessions in alternating sequences. This resulted in remission in all four MDD or BD patients. Of interest, all patients suffered from severe TRD, including non-response to prior ECT. These results indicate that repetitive IV infusions might be able to augment the anti-depressive effect of ECT.

In summary, in the available repeated IV infusion studies, a clinical response to esketamine was maintained over the course of treatment, and for up to 35 days afterwards. Response was even observed in TRD patients with prior non-response to ECT after alternating ECT with esketamine IV infusions.

##### SC injections

SC injections of esketamine were provided in four studies published in seven articles (Ajub and Lacerda, 2018; Barbosa et al., 2020; Del Sant et al., 2020; Delfino et al., 2021; Findeis et al., 2020; Lucchese et al., 2021; Ritter et al., 2020).

Of the three patients with depression with psychotic features described by Ajub and Lacerda (2018), two received a single 0.5 mg/kg SC injection of esketamine. This resulted in remission of both depressive and psychotic symptoms at 24 hours, which was maintained for up to 4 weeks follow-up.

A second study reported on the positive effects of four SC injections of up to 0.75 mg/kg esketamine in a patient with severe MDD and a metastatic abdominal tumor (Barbosa et al., 2020).

In a larger retrospective case series in 70 MDD and BD patients, SC injections were given weekly for 6 weeks in doses up to 1.0 mg/kg. Patients met responder and remission criteria in 50% and 26%, respectively (Del Sant et al., 2020; Delfino et al., 2021; Lucchese et al., 2021).

Findeis et al. (2020) and Ritter et al. (2020) reported on a therapeutic regimen combining a single IV infusion with twice or thrice weekly SC injections of up to 0.5 mg/kg esketamine. In the sample of 25 MDD, BD, and schizoaffective disorder patients, responder and remission criteria were met in 31% and 45%, respectively.

Summarized, these results suggest a rapid onset of antidepressant effectiveness after a single SC injection of esketamine, and robust antidepressant effectiveness over the course of treatment with repeated SC injections.

##### Oral administration

Two studies have assessed the antidepressant effects of oral esketamine (Paslakis et al., 2010; Veraart et al., 2021a).

The four MDD patients described by Paslakis et al. (2010) received 1.25 mg/kg esketamine for 12–14 days, as add-on to a recently started standard antidepressant medication. While two patients did not respond to the combined treatment, the other two did.

An MDD patient who suffered from severe TRD, including non-response to prior ECT and deep brain stimulation, received 2.0 mg/kg esketamine twice weekly for over 18 months. This resulted in remission at 6 weeks, which was maintained over the course of treatment (Veraart et al., 2021a).

Overall, these preliminary results indicate potential long-term antidepressant effects of repeated treatment with oral esketamine in patients with MDD and severe TRD.

More details are provided in Table 2.

### Safety

#### Randomized controlled trials

Acute psychiatric adverse events were assessed in one trial, showing a dose-dependent peak at 30 to 40 min after IV infusion started and a return to baseline within 2 h (Singh et al., 2016). Psychotomimetic adverse events were assessed in two trials, showing a similar dose-dependent pattern and no differences between esketamine and racemic ketamine groups (Correia-Melo et al., 2020; Singh et al., 2016). Dissociation was the only cited adverse event causing withdrawal (in one patient) (Singh et al., 2016).

The most common neurological adverse events were dizziness and headache. Rates were comparable between esketamine, racemic ketamine, and placebo groups (Liu et al., 2020; Singh et al., 2016; Wang et al., 2020).

The most common gastrointestinal adverse events were nausea and vomiting. Again, rates were comparable between the three groups (Liu et al., 2020; Singh et al., 2016; Wang et al., 2020).

Vital functions were assessed in two trials (Correia-Melo et al., 2020; Singh et al., 2016). According to Singh et al. (2016), no clinically significant vital sign abnormalities were observed with esketamine, except for one case of transient irregular breathing and one case of transient high blood pressure. According to Correia-Melo et al. (2020) increased blood pressure (BP) and heart rate (HR) were mild, self-limiting, and equally distributed among the esketamine and ketamine patients.

Other cited adverse events of esketamine were dry mouth (*n* = 3), nasopharyngitis (*n* = 2), oropharyngeal pain (*n* = 2), paresthesia (*n* = 2), vertigo (*n* = 2), rash (*n* = 1), and thrombophlebitis (*n* = 1) (Singh et al., 2016). The only reported serious adverse event (cancer) occurred during the post-treatment phase and was considered unrelated to esketamine treatment (Singh et al., 2016).

In summary, these results indicate that esketamine elicits dose-dependent and transient acute psychiatric and psychotomimetic adverse events, and transient increased BP and HR. When compared to ketamine, these adverse events were generally found to be similar in frequency and intensity. No treatment-related serious adverse events have occurred, and withdrawal due to adverse events was limited to one case of the 813 patients included in the RCTs. More details are provided in Table 1.

#### Open-label studies

The most common adverse events were psychotomimetic in nature and were reported in 7 of 15 studies (Ajub and Lacerda, 2018; Barbosa et al., 2020; Bartova et al., 2018; Correia-Melo et al., 2017a, 2017b; Paul et al., 2009; Segmiller et al., 2013). These psychotomimetic effects were generally mild and did not persist long after administration, except for three cases. Correia-Melo et al. (2017b) described two cases with severe psychomimetic effects that were experienced as traumatic. These effects occurred with rapid (10 min) infusion of esketamine. Segmiller et al. (2013) described one case with severe dissociation associated with 40-min IV infusion of 0.25 mg/kg esketamine, leading to treatment discontinuation. Noteworthy, in the case series involving esketamine as well as racemic ketamine, both patients experienced psychomimetic adverse events during racemic ketamine but not esketamine infusion. (Paul et al., 2009). Also of interest is that no induction or worsening of positive psychotic symptoms was observed in patients with depression with psychotic features, schizophrenia, or schizoaffective disorder (Ajub and Lacerda, 2018; Bartova et al., 2018; Findeis et al., 2020; Veraart et al., 2021a).

Other adverse events reported were nausea, dizziness/light-headedness, confusion, disorientation, fatigue, feeling “muzzy,” intrusions, increased anxiety, and unintentional crying (Ajub and Lacerda, 2018; Kavakbasi et al., 2021; Paul et al., 2009; Ritter et al., 2020; Veraart et al., 2021a). Furthermore, isolated cases were reported of recurrent headaches, upper respiratory infection, carious tooth burst, transient worsening of lower back pain, and worsening of abdominal pain. The latter were “considered unrelated to esketamine treatment” according to the authors (Barbosa et al., 2020; Kallmünzer et al., 2016).

Vital functions were reported in eight studies (Barbosa et al., 2020; Bartova et al., 2015, 2018; Correia-Melo et al., 2017a; Del Sant et al., 2020; Paul et al., 2009; Ritter et al., 2020; Veraart et al., 2021a). Cardiovascular changes were mostly “within normal ranges” or “not relevant” according to the authors. Ritter et al. (2020) reported on a single case with transient hypertension. Del Sant et al. (2020) specifically aimed to assess the cardiovascular safety of repeated SC injections of esketamine. Maximum mean BP levels were reached within 30–45 min. Treatment emergent transient hypertension (systolic BP (SBP) ⩾ 180 and/or diastolic BP (DBP) ⩾ 110) was found in 20% of patients. Within 2 h, both SBP and DBP returned to pre-dose levels. HR did not show significant differences throughout any treatment session.

To assess whether esketamine treatment is associated with urinary toxicity, urine samples were analyzed in one case series. Leukocyte, erythrocyte, free hemoglobin, and protein concentrations did not display a rise over the course of up to 34 esketamine administrations, suggesting absence of short-term urothelial damage (Findeis et al., 2020).

Withdrawal and lost-to-follow-up were reported in four studies, of which two (published in three articles) specified withdrawal due to adverse events (Findeis et al., 2020; Ritter et al., 2020; Segmiller et al., 2013). Ritter et al. (2020) and Findeis et al. (2020) reported a withdrawal rate due to adverse events of 17% (*n* = 5) and 8% (*n* = 2), respectively, without further specification. The only specified reason for withdrawal was dissociation after 0.25 mg/kg IV infusion of esketamine (*n* = 1) (Segmiller et al., 2013).

In summary, the open-label data on the safety of esketamine are generally in agreement with the RCT data. Three types of adverse events were identified that were not reported in the RCTs: disorientation, fatigue, and increased anxiety. In addition, the open-label safety data indicate marked psychotomimetic symptoms in exceptional cases. They do not indicate induction or worsening of positive psychotic symptoms in predisposed patients, nor immediate urinary toxicity. More details are provided in Table 2.

## Discussion

Although studies have shown IV ketamine and IN esketamine to be effective treatment strategies for many TRD patients, it is needed to continue studying alternatives for several reasons, including reasons of availability, costs, and optimal (sustained) response, safety, and tolerability. To our knowledge, this is the first systematic review of the literature on the antidepressant effect and safety of non-intranasal esketamine for depression. The combined results of 19 studies, describing treatment of 981 patients across 24 articles, suggest that intravenous, subcutaneous, and possibly oral esketamine are effective and that adverse events are mostly mild and transient. Therefore, these non-intranasal esketamine options may offer a valuable addition to the depression treatment armamentarium.

### Antidepressant effects

We found IV, SC, and possibly oral esketamine to be effective in reducing depressive symptoms on the short term and over the course of treatment. Moreover, response was not only observed in patients with MDD, but also in patients with BD and severe TRD. Particularly in the open-label studies, many patients had severe and chronic depression and high levels of treatment refractoriness, including for ECT. This usually predicts a poor response to subsequent treatment, but esketamine treatment showed antidepressant effects nevertheless.

An additional observation worth mentioning, is that some reviewed studies indicate that esketamine may be at least as effective in reducing depressive symptoms as racemic ketamine. Although data are preliminary, this is important, as the availability and costs of ketamine and esketamine show substantial variation between countries. However, recent findings also suggest that IV racemic ketamine is superior to IN esketamine in both response and remission rates (Bahji et al., 2021). In the absence of studies directly comparing the efficacy of these two, firm conclusions regarding comparative efficacy cannot yet be drawn (Drevets et al., 2021). This is also the case for comparisons between different routes of administration of esketamine.

When comparing the short-term results of previous IN esketamine RCTs (Papakostas et al., 2020; Zheng et al., 2020) to the short-term results of the two included non-intranasal RCTs (both IV) that are the most comparable in terms of patient populations, it appears response rates are higher for IV esketamine compared to IN esketamine (i.e. 50% to 67% versus 21% after the first esketamine administration). At the same time, however, remission rates might be of the same order (i.e. approximately 30%), suggesting the two routes may yield comparable clinical outcomes at the short-term. Clearly, caution should be exercised, as direct comparisons have not been made. In addition, the efficacy of SC and oral esketamine is even harder to compare to the efficacy of IN esketamine, as no RCTs studying these two routes have been conducted yet.

### Safety

Most adverse events were found to be mild and had resolved shortly after esketamine administration. No treatment-related serious adverse events have occurred in the RCTs, and withdrawal due to adverse events was limited to one case. In general, the open-label safety data are in agreement with the RCT safety data, which showed no treatment-related serious adverse events. Nonetheless, they indicate marked psychotomimetic symptoms in exceptional cases and identified three types of adverse events that were not reported in the RCTs: disorientation, fatigue, and increased anxiety. These symptoms as well as marked psychomimetic symptoms have, however, also been reported after racemic ketamine and IN esketamine administration (Fedgchin et al., 2019; Short et al., 2018; Wajs et al., 2020).

Two additional findings are worth mentioning. First, our review does not support the assumption that esketamine induces or worsens positive psychotic symptoms in predisposed patients. This is in line with the findings of a recent systematic review on ketamine for depression in patients with a history of psychosis or current psychotic symptoms (Veraart et al., 2021b), and has important clinical implications, as psychotic features are common in depressed patients (Jääskeläinen et al., 2018). Second, preliminary results indicate that esketamine is unlikely to cause urothelial toxicity on the short-term. This is essential given the potential urological toxicity of (es)ketamine.

Overall, our review indicates that esketamine is well tolerated by most patients and demonstrates a safety pattern comparable to racemic ketamine and IN esketamine. However, again, firm conclusions regarding comparative safety and tolerability cannot yet be drawn.

### Limitations

Some limitations in this review need to be addressed. First, patient populations, treatment regimens, and outcome definitions were not the same across the included studies, limiting the comparability of results. Second, the quality of the studies varied considerably. The overall quality of the RCTs and case–control study was considered high, but most case reports and series had high risks of bias. Besides, a major limitation of most studies was the inadequacy of active and structured inquiry of adverse events.

While the CADSS and cardiovascular measures were used in several studies, other categories of adverse events were often not specifically assessed, including psychiatric, neurological, cognitive, gastro-intestinal, and urological adverse events. This is important in view of previous findings suggesting that these adverse events occur, and that repeated use of ketamine in recreational users is linked with urological toxicity, hepatotoxicity, cognitive deficits, and dependency risks (Short et al., 2018). Other major limitations of most studies were the lack of long-term follow-up assessments and small sample sizes.

When comparing the esketamine doses that are used in the IV studies described in this review to the racemic ketamine doses that are used in the majority of previous IV studies, it is noticeable that there is more variation and a wider range in esketamine dosing. IV ketamine doses tend toward a standard 0.5 mg/kg. By contrast, IV esketamine was administered in 10 different doses, ranging from 0.125 mg/kg to 1.0 mg/kg. Research has shown equivalent clinical effects with a 2:1 racemic to esketamine dosing on electroencephalography (Ihmsen et al., 2001) and in surgical anesthesia (Geisslinger et al., 1993). It is not clear if this ratio is also applicable to IV (es)ketamine in the treatment of depression. Regarding SC and oral administration, doses vary widely between both esketamine and racemic ketamine studies. This could suggest that optimal SC and oral dosing is not yet known, but also that individual dosing is preferred.

### Future directions

The concept of NMDA receptor antagonism has been challenged, and various other molecular insights have been gained in the mechanistic pathways of ketamine and its enantiomers (Jelen et al., 2021). This offers perspective for alternatives, like arketamine. Previously it was assumed that the undesired psychic emergence reactions of ketamine were associated with arketamine. However, existing data on this point are still a subject of controversial discussion. Better tolerability of esketamine than racemic ketamine was demonstrated in earlier studies in both rodents (Liu et al., 2006) and humans (Muller et al., 2016; Pfenninger et al., 2002; White et al., 1980). For this reason, esketamine is widely used in anesthesia. Conversely, others reported that sub-anesthetic doses of arketamine induced a state of relaxation and feeling of well-being, while in the same individuals a sub-anesthetic dose of esketamine induced psychotomimetic effects (Passie et al., 2021; Vollenweider et al., 1997). A further matter is that these “undesired psychic emergence reactions” of ketamine may also help in the psychotherapeutic process (Dore et al., 2019; Luckenbaugh et al., 2014). This points to a need to also compare effects of arketamine and esketamine in patients with depression.

In theory, esketamine has the potential to have a superior antidepressant effect and safety profile compared to racemic ketamine, but current evidence is not sufficiently robust to confirm this hypothesis. Adequately powered comparative studies are needed, focusing on both the short- and long-term efficacy and safety of different types and formulations of ketamine. Time will tell whether non-intranasal esketamine may offer an effective and safe addition to our depression treatment armamentarium.

## Supplemental Material

sj-docx-1-jop-10.1177_02698811221084055 – Supplemental material for The antidepressant effect and safety of non-intranasal esketamine: A systematic review Click here for additional data file. Supplemental material, sj-docx-1-jop-10.1177_02698811221084055 for The antidepressant effect and safety of non-intranasal esketamine: A systematic review by Sanne Y Smith-Apeldoorn, Maurice Vischjager, Jolien KE Veraart, Jeanine Kamphuis, Marije aan het Rot and Robert A Schoevers in Journal of Psychopharmacology

sj-docx-2-jop-10.1177_02698811221084055 – Supplemental material for The antidepressant effect and safety of non-intranasal esketamine: A systematic review Click here for additional data file. Supplemental material, sj-docx-2-jop-10.1177_02698811221084055 for The antidepressant effect and safety of non-intranasal esketamine: A systematic review by Sanne Y Smith-Apeldoorn, Maurice Vischjager, Jolien KE Veraart, Jeanine Kamphuis, Marije aan het Rot and Robert A Schoevers in Journal of Psychopharmacology
