# Supplementary material for: The antidepressant effect and safety of non-intranasal esketamine: A systematic review
Source: J Psychopharmacol. 2022 May 12;36(5):531–44. doi: 10.1177/02698811221084055 (PMC9112628; doi:10.1177/02698811221084055)
Supplement: sj-docx-1-jop-10.1177_02698811221084055 – Supplemental material for The antidepressant effect and safety of non-intranasal esketamine: A systematic review [file sj-docx-1-jop-10.1177_02698811221084055.docx]

**Supplementary Table 1.** Search strategy.

| **Database** | **Search string** |
| --- | --- |
| PubMed | ("Bipolar Disorder"[Mesh] OR "Mood Disorders"[Mesh] OR bipolar*[tiab] OR depress*[tiab] OR unipolar*[tiab] OR mood*[tiab] OR affective*[tiab]) AND ("Esketamine" [Supplementary Concept] OR esketamine[tiab] OR s-ketamine[tiab]) |
| Embase | ('mood disorder'/exp OR bipolar*:ab,ti OR depress*:ab,ti OR unipolar*:ab,ti OR mood*:ab,ti OR affective*:ab,ti) AND (esketamine/exp OR esketamine:ab,ti OR ‘s-ketamine’:ab,ti) |
| Cochrane Library | (bipolar* OR mood* OR unipolar* OR depress* OR affective*) AND (esketamine OR s-ketamine) |
| Google Scholar | allintitle: esketamine OR s-ketamine depression OR depressive OR mood OR affective OR bipolar OR unipolar |
