# Supplementary material for: The antidepressant effect and safety of non-intranasal esketamine: A systematic review
Source: J Psychopharmacol. 2022 May 12;36(5):531–44. doi: 10.1177/02698811221084055 (PMC9112628; doi:10.1177/02698811221084055)
Supplement: sj-docx-2-jop-10.1177_02698811221084055 – Supplemental material for The antidepressant effect and safety of non-intranasal esketamine: A systematic review [file sj-docx-2-jop-10.1177_02698811221084055.docx]

**Supplementary Table 2.** Risk of bias assessment of RCTs according to the Cochrane risk-of-bias tool for randomized trials.

**Supplementary Table 3.** Risk of bias assessment of case reports and series according to the Quality Appraisal of Case Series Studies Checklist.

|  | Objective | Design | | Population | | (Co-)Intervention | | Outcome | | | Results | Declaration |
| --- | --- | --- | --- | --- | --- | --- | --- | --- | --- | --- | --- | --- |
|  | Clear aim | Prospective | Consecutive recruiting | Relevant characteristics described | Clear eligibility criteria | Clear intervention | Clear co-intervention | Blinded assessors | Appropriate methods | Before and after measures | Follow-up long enough | Competing interests and support |
|  |  |  |  |  |  |  |  |  |  |  |  |  |
| Ajub 2018 | N | U | U | P | N | Y | Y | N | P | P | Y | P |
| Barbosa 2020 | Y | U | U | Y | N | Y | Y | N | Y | P | Y | Y |
| Bartova 2015 | Y | U | U | P | N | P | Y | N | N | U | U | P |
| Bartova 2018 | N | U | U | P | N | P | Y | N | Y | P | U | Y |
| Correia-Melo 2017a | Y | N | U | Y | Y | Y | Y | N | P | P | N | P |
| Correia-Melo 2017b | Y | U | U | Y | N | Y | Y | N | P | Y | Y | P |
| Del Sant 2020 | Y | N | U | P | Y | Y | Y | N | Y | Y | Y | Y |
| Delfino 2020 | Y | N | Y | P | Y | Y | P | N | P | Y | N | Y |
| Findeis 2020 | Y | N | U | P | Y | P | N | N | Y | Y | Y | Y |
| Kallmünzer 2016 | P | Y | U | Y | N | Y | Y | N | P | Y | Y | N |
| Kavakbasi 2020 | Y | U | U | Y | N | Y | P | N | P | P | N | Y |
| Lucchese 2021 | Y | N | Y | Y | Y | Y | Y | N | Y | Y | N | Y |
| Paslakis 2010 | Y | U | U | P | N | Y | Y | N | P | P | N | N |
| Paul 2009 | Y | U | U | Y | N | Y | Y | N | P | P | Y | P |
| Ritter 2020 | N | U | U | P | N | P | P | N | P | Y | N | P |
| Segmiller 2013 | Y | U | U | Y | N | Y | Y | N | P | P | N | P |
| Veraart 2021 | Y | Y | U | Y | N | Y | Y | N | P | P | Y | Y |

N, no; P, partial; U, unclear; Y, yes

**Supplementary Table 4.** Risk of bias assessment of case-control studies according to the Newcastle-Ottawa Scale.

|  | Selection | | | | Comparability | Exposure | | |
| --- | --- | --- | --- | --- | --- | --- | --- | --- |
|  | Adequate definition | Representativeness of cases | Selection of controls | Definition of controls | Comparability of cases and controls | Ascertainment of exposure | Comparability of ascertainment | Non-response rate |
| Falk 2020 | Y | Y | Y | Y | Y | Y | Y | U |

U, unclear; Y, yes
